# Automatic Artifact Removal from Electroencephalogram Data Based on A Priori Artifact Information

**DOI:** 10.1155/2015/720450

**Published:** 2015-08-25

**Authors:** Chi Zhang, Li Tong, Ying Zeng, Jingfang Jiang, Haibing Bu, Bin Yan, Jianxin Li

**Affiliations:** China National Digital Switching System Engineering and Technological Research Center, Zhengzhou 450002, China

## Abstract

Electroencephalogram (EEG) is susceptible to various nonneural physiological artifacts. Automatic artifact removal from EEG data remains a key challenge for extracting relevant information from brain activities. To adapt to variable subjects and EEG acquisition environments, this paper presents an automatic online artifact removal method based on a priori artifact information. The combination of discrete wavelet transform and independent component analysis (ICA), wavelet-ICA, was utilized to separate artifact components. The artifact components were then automatically identified using a priori artifact information, which was acquired in advance. Subsequently, signal reconstruction without artifact components was performed to obtain artifact-free signals. The results showed that, using this automatic online artifact removal method, there were statistical significant improvements of the classification accuracies in both two experiments, namely, motor imagery and emotion recognition.

## 1. Introduction

As a biological signal that reflects potential changes in complex brain activities, electroencephalogram (EEG) plays an important role in human brain research, disease diagnosis, brain-computer interfaces (BCI), and so on. However, the electrical signals of brain activities are weak, so real EEG is susceptible to various nonneural physiological artifacts. The most severe artifacts include eye movement (electrooculography, EOG) and muscle movement (electromyography, EMG) artifacts [1]. These undesired signals can complicate EEG data or can be misread as the physiological phenomena of interest. Thus, eliminating the effects of artifacts and extracting the most relevant information from brain activities are key challenges for researchers.

Artifact avoidance and artifact rejection were used to handle artifacts in early studies. These approaches might not acquire sufficient valid data from actual experiments, in which eye blinking, swallowing, or other nonneural physiological activities are inevitable [2]. Linear filtering is an advanced method that may be used, but it is not recommended especially when neural signals of interest are in the same frequency range as that of artifacts [3]. Linear regression [4, 5] assumes that EEG measurement is a linear combination of real EEG and artifacts and they are not related. This straightforward technique works well for EOG artifacts with a reference channel, but the assumption is inadequate for removing EMG artifacts. A more extensive review of artifact reduction techniques can be obtained from the literature [1].

Blind signal separation (BSS) techniques are the most promising approach for separating the recordings into components that “build” them. They regard EOG, EMG, and other artifacts as the signals produced by independent sources. BSS techniques need to identify components that are attributed to artifacts and perform signal reconstruction without them [6]. Independent component analysis (ICA) is a widely used BSS method. ICA was first applied in routine EEG analysis by Makeig et al. [7] in 1996. EOG [8] and EMG [9] artifacts can be successfully separated from EEG signals. Flexer et al. [10] proved that the irregular EOG artifact of the blind can also be separated by ICA. Several studies have also used ICA technique for artifact removal [11–19].

Automatic artifact removal from EEG is preferred in practice. It is suitable for only EOG artifact removal with a reference channel [8, 18]. A concept similar to placing an accelerometer on the head is applied to head movement artifact removal [19]. To remove EOG and EMG artifacts simultaneously, researchers prefer to combine machine learning in their automatic systems. Previous studies combined BSS/ICA and support vector machine (SVM) to automatically remove artifacts [16, 17]. Winkler et al. [20] used a linear programming machine to automatically classify general artifactual source components. However, machine learning processes require many offline training samples, which need to be visually inspected and manually labeled as different artifacts. Furthermore, offline trained classifiers may not perform optimally for variable subjects and EEG acquisition environments. Accordingly, an effective solution is to distinguish artifact components automatically, easily, and accurately during a single acquisition.

This paper proposes a novel automatic artifact removal method for variable subjects and EEG acquisition environments. Without reference channels and massive offline training samples, a small amount of time is used to acquire individual artifact samples as online a priori artifact information in advance. Automatic identification and removal of artifact components are realized using correlation analysis and wavelet-ICA (WICA). At last, the method is applied to two classification experiments, namely, motor imagery and emotion recognition. The experimental results showed that there were statistical significant improvements of the classification accuracies by applying this automatic online artifact removal method.

## 2. Method

The following subsections describe how the proposed automatic artifact removal approach was established. We also applied the approach to two classification experiments, namely, motor imagery and emotion recognition.

### 2.1. Online Extraction of A Priori Artifact Information

**Figure 1:**
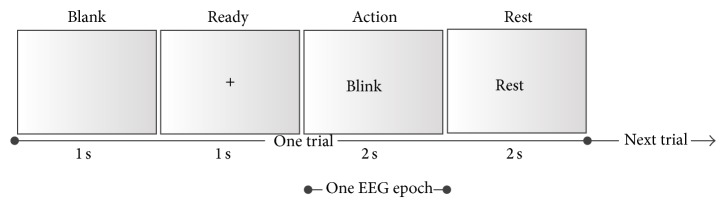
One trial of the artifact acquisition session. One trial consists of one 1 s blank period, one 1 s ready period, one 2 s action period, and one 2 s rest period. When a visual cue is presented in the action period, the subjects are required to do the corresponding action only. The EEG epoch represents the data used for the following automatic artifact removal.

We first describe how to obtain a priori artifact information online, which is necessary for the following automatic artifactual component identification. During the actual EEG acquisition, artifacts are often generated by the movements of subjects, intentionally or unintentionally, such as eye blinking, eye rolling, teeth clenching, and swallowing. If the subject does only one action for a time period, the corresponding recoding data can be clearly marked by a corresponding artifact label, which can be utilized for the following automatic artifact classification. Thus, an artifact acquisition session was performed to extract a priori artifact information before the formal EEG data acquisition.  Figure 1 shows a trial of the experimental design. At the beginning of one trial was a 1 s blank period, followed by a 1 s ready period, in which subjects were instructed to stare at the center fixation cross and try not to think of anything on purpose. A visual cue appeared for 2 s to indicate the corresponding action to the subjects. Given that typical actions can arouse artifacts, eye blinking, eye rolling, and teeth clenching were chosen as stimuli. No movement was set as the control stimulus. When the visual cue was presented in the screen, the subjects were required to do the corresponding action only. A 2 s rest period ended the trial. The artifact acquisition session contained 40 trials, with 10 trials for each type of stimulus. The total time was 240 s.

### 2.2. Automatic Artifact Removal Using A Priori Artifact Information and WICA

As a combination of DWT and ICA, WICA is proposed based on the joint use of multiresolution and multidimensional analyses. WICA was first proposed for the processing of EMG signals [21, 22]. WICA improves the performance of ICA because it projects data into a new space where the redundancy is higher and the features of artifacts are fully utilized [23]. The coefficients of wavelet transform exhibit a more super-Gaussian nature in the probability density function and larger kurtosis than the raw signal. Li et al. [12] implemented automatically EOG artifact reduction using a reference channel and WICA and proved that WICA greatly improves the SNR of EEG signals and antinoise capability.

This paper presents a novel WICA technique optimized for automatic EOG and EMG artifact removal using individual a priori artifact information acquired online in advance. This section explains how the proposed method allows automatic EOG and EMG artifact removal.

**Figure 2:**
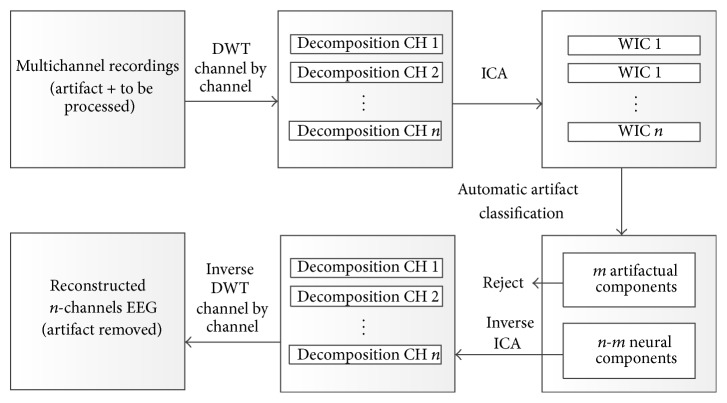
Block diagram of WICA for automatic EEG artifact removal. Raw data to be removed are appended to the artifact samples first. The next stage is wavelet decomposition via channel by channel, in which data are projected into *n*-dimensional space where ICA is performed. Subsequently, *n*-*m* neural-related WICs are used for *n*-channel wavelet coefficient reconstruction, whereas *m* artifactual WICs are automatically recognized by correlation analysis. Finally, the *n*-channel EEG signal without artifacts is reconstructed by inverse DWT from *n*-channel wavelet coefficient.

The algorithm for EOG and EMG artifact removal in EEG (as shown in Figure 2) is presented as follows.

(1) Raw data to be processed were appended to the artifact samples first. Mallat's pyramid decomposition algorithm was applied to *n*-channels of signals (artifact samples + to be processed):

(1) $$\begin{array}{c} X\left(t\right)={\left[x_{1}\left(t\right),x_{2}\left(t\right),\ldots x_{n}\left(t\right)\right]}^{T}. \end{array}$$

Each channel *x*_*i*_(*t*) ∈ *R* ^*M*_1_×1^  (*i* = 1,2,…, *n*) was decomposed by *L*-level decomposition tree, and the approximate coefficients and detail coefficients were ranked to construct the wavelet coefficient vector $\vec{u_{i}}\in R^{M_{2}\times 1}$:

(2) $$\begin{array}{c} \vec{u_{i}}={\left[A_{i,L},D_{i,L},D_{i,L-1},\ldots ,D_{i,1}\right]}^{T},\;i=1,2,\ldots ,n. \end{array}$$

(2) All coefficient vectors

(3) $$\begin{array}{c} U={\left[\vec{u_{1}},\vec{u_{2}},\ldots ,\vec{u_{n}}\right]}^{T} \end{array}$$

were combined and considered the input of the ICA algorithm. The FastICA algorithm based on a negentropy criterion was applied to estimate the separation matrix *W* ∈ *R* ^*n*×*n*^, and the *n*-channels of independent wavelet-domain components

(4) $$\begin{array}{c} Y={\left[\vec{y_{1}},\vec{y_{2}},\ldots ,\vec{y_{n}}\right]}^{T} \end{array}$$

were obtained using the following equation:

(5) $$\begin{array}{c} Y=WU. \end{array}$$

(3) The a priori artifact information and correlation analysis were applied to recognize EOG and EMG wavelet-independent components (WICs). First, Mallat's pyramid construction algorithm was applied to each WIC $\vec{y_{i}}\in R^{M_{2}\times 1}$ to reconstruct the corresponding time-domain component $\vec{v_{i}}\in R^{M_{1}\times 1}$, which was as long as the original signal *x*_*i*_(*t*) ∈ *R* ^*M*_1_×1^. For each artifact EEG epoch, Welch's algorithm was applied to calculate the power spectrum density (PSD) from 1 Hz to 50 Hz. Subsequently, the PSD was used to calculate the relative energy in 10 Hz-wide frequency bins, thereby yielding five bins. We then used the correlation score (the absolute value of Pearson correlation coefficient, |*r*|) to represent the correlation between the five PSD features of each component and artifact labels. Spectral information is particularly helpful for the classification of artifacts because EOG and EMG have different typical spectra. That is, EOG artifacts show much more energy at lower frequencies, whereas EMG generally contaminates all the frequency ranges of interest. For the corresponding artifact components, the correlation score of the corresponding frequency band was the highest. Instead of visually inspecting or using reference channels, we recognized corresponding EOG and EMG WICs using the proposed method. EMG artifacts tend to contaminate most components with varying degrees of intensity, and recognizing every component with only traces of EMG as artifact can result in the excess removal of nonartifact EEG data [17]. Therefore, only those components with strong EMG were recognized as EMG artifacts, which was easily accomplished by ranking the correlation coefficients. All the steps described in the artifact identification process for all components were performed automatically by computer.

(4) Entity matrix *E* was constructed, where *E*_(*i*,*i*)_ = 0 if the component *i* was recognized as an artifact. The *n*-channels of WICs $Y={\left[\vec{y_{1}},\vec{y_{2}},\ldots ,\vec{y_{n}}\right]}^{T}$ were projected back onto the scalp electrodes with inverse transform of ICA using the following equation:

(6) $$\begin{array}{c} Z=W^{-1}EY,\;Z\in R^{n\times M_{2}}. \end{array}$$

(5) Mallat's pyramid construction algorithm was applied to each channel of wavelet coefficients *Z* to reconstruct the artifact-free EEG data. Thus, the EOG and EMG artifacts in the EEG signals were removed.

### 2.3. Validation 1: Application to Motor Imagery

In this section, we applied our automatic artifact removal approach to a motor imagery classification experiment. We investigated the effects of our approach on the classification performance of motor imagery based on PSD.

#### 2.3.1. Experimental Setup

**Figure 3:**
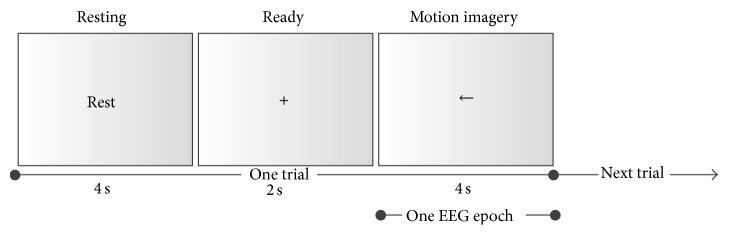
One trial of the motor imagery experiment. The EEG epoch represents the data used for analysis and classification.

Fourteen healthy BCI novices performed first motor imagery with the left hand, right hand, and neither in a calibration measurement without feedback. Every 10 s, one of three different visual cues (arrows pointing left, right, or both) indicated to the subject which type of motor imagery to perform (Figure 3). Twenty trials of each motor condition were recorded in random order. The sessions were recorded using a 16-channel g.USBamp system (Table 1). The recordings were conducted at a sampling frequency of 512 Hz using an activated high-pass filter at 0.1 Hz, low-pass filter at 60 Hz, and notch filter at 50 Hz to suppress power line noise. A Priori artifact information was acquired in advance during artifact acquisition sessions and then incorporated in our automatic artifact removal approach.

#### 2.3.2. Data Analysis

After artifact removal, data from three electrodes (C3, CZ, and C4) in the motion imagery period were selected to extract the power spectrum feature for the input of SVM classification. First, the 4 s long epoch was equally divided into four segments. Second, each segment of the EEG data was processed with the Hanning window. Third, windowed segments were extended by zero padding for fast Fourier transform. Finally, EEG power spectra were extracted in 45 bands from 1 Hz to 45 Hz, and each band was 1 Hz long. Thus, the total number of feature dimensions was 540.

Correlation-based feature selector is a type of supervised dimensionality reduction method [24]. Each feature obtains a score presenting its correlation with a label by this method. The most label-relevant features can be found by ranking these scores. The selected features were used for RBF-kernel SVM classification. To validate our automatic artifact removal approach, we also analyzed the data without artifact removal in the same manner.

### 2.4. Validation 2: Application to Emotion Recognition

To evaluate the artifact removal performance for EEG data from higher-order cognitive processes, data from thirteen healthy subjects were used to test the proposed automatic artifact removal method in another classification experiment, namely, an emotion recognition study.

#### 2.4.1. Experimental Setup

Thirteen college participants aged 20 to 24 years with normal or corrected-to-normal vision participated in this study. All participants had no neurological or psychological medical history. Before experiments, we obtained informed consent from each participant. The pictures used for emotion induction were obtained from the Chinese Affective Picture System [25]. These pictures were all rated in terms of the valence and arousal levels. They were divided into five categories, namely, very high valence (VHV), high valence (HV), neutral, low valence (LV), and very low valence (VLV). ANOVA showed that the categories were significantly different in the valence level (*P* < 0.05) but not significantly different in the arousal level (*P* > 0.05).

**Figure 4:**
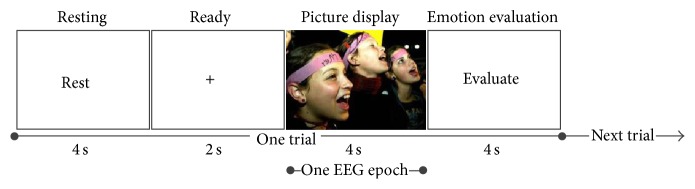
One trial of the emotion recognition experiment. The EEG epoch represents the data used for analysis and emotion classification.

The emotion induction experiment (150 trials) is illustrated in Figure 4. Each trial started with a 4 s resting period, followed by a 2 s ready period, during which subjects were instructed to stare at the center fixation cross and try not to think of anything on purpose. Subsequently, a picture was presented to the subjects for 4 s, during which participants were instructed to try to engage themselves into the emotion represented by the picture. At the end of each trial, participants were given 4 s to evaluate the perceived emotion and categorize it as one of the five categories by pressing number keys 1 to 5. The acquisition equipment and parameter settings were similar to those used in Validation 1.

#### 2.4.2. Data Analysis

Power spectrum features are widely used for emotion recognition because they can be analyzed to characterize the perturbations in the oscillatory dynamics of ongoing EEG [26, 27]. In this data analysis, all 16 channels of EEG signals in the picture display period were used to extract the power spectrum feature. We calculated the power spectrum feature for each 1 Hz (from 1 Hz to 45 Hz) and each 1 s. Thus, the feature vector for each trial with 2880 dimensions was obtained using the same method in Validation 1. Finally, the features selected by correlation-based feature selector were used for RBF-kernel SVM classification. The labels of EEG epochs for classification were determined by participants' subjective psychometric evaluation. We also analyzed the data without artifact removal in the same manner to validate our automatic artifact removal approach.

## 3. Results 

In the following subsections, we investigated the validity of our method via correlation analysis for identifying artifact components. We also comparatively analyzed the signal waveform and classification performances of two validation experiments before and after artifact removal.

### 3.1. Performance of Correlation Analysis for Identifying Artifact Components

**Figure 5:**
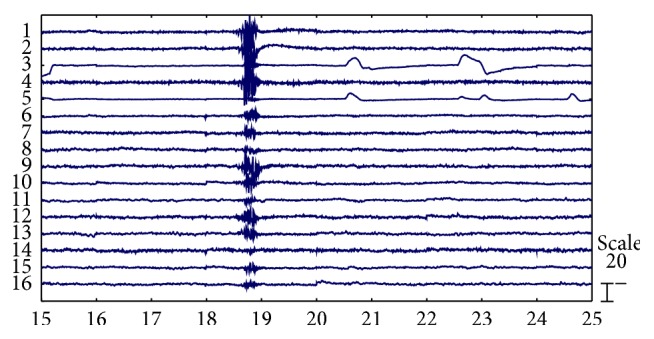
Corresponding time-domain components of the WICs.

**Figure 6:**
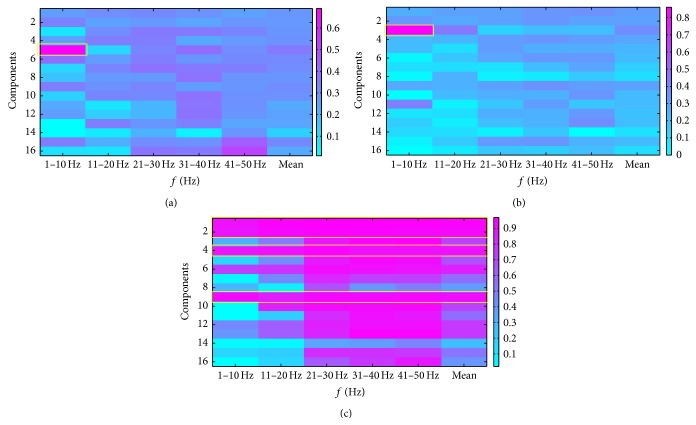
Correlation scores (|*r*|) plots between five PSD features of each component and artifact labels. Plots (a), (b), and (c) show the correlation scores with eye blinking, eye rolling, and teeth clenching, respectively. Regions of interest are marked with yellow boxes.

We plotted the corresponding time-domain components of the WICs to visually inspect and identify the artifacts and compared the results of automatic identification by correlation analysis.  Figure 5 shows the corresponding time-domain components of the WICs. Components 3 and 5 represent EOG artifacts, whereas components 1, 2, 4, and 9 are intuitively considered artifacts containing strong EMG.  Figure 6 shows the correlation scores between five PSD features of each component and artifact labels. By ranking all the correlation coefficients, we found that the EOG artifact components exhibited the highest correlation scores (1–10 Hz) with eye blinking label and eye rolling label. Similarly, a number of strong EMG artifact components were recognized by ranking correlation scores with teeth clenching label. We selected the four highest mean scores as the EMG artifacts to be removed. By comparison, we found the artifact components that the highest correlation scores represented were the same as those we visually inspected. Therefore, all artifact components could be automatically recognized by correlation analysis.

From another perspective, the different distributions of correlation scores between EOG and EMG may indicate the different characteristic power spectrum between them. EOG artifact components showed significantly higher energy in low power spectrum (1–10 Hz) (Figures 6(a) and 6(b)), whereas EMG artifacts were almost distributed in all the power spectra (1–50 Hz) (Figure 6(c)). Notably, EMG artifacts almost disturbed all the components (Figure 5), so EMG artifact removal in EEG remains a serious challenge. This problem may be the reason why few studies about artifact removal have studied the automatic method for the removal of EMG artifacts [1].

We implemented our method in Matlab 2012b. The average computation cost of the automatic artifact removal method for one single trial was about 5 s. In it, The DWT and ICA accounted for a higher proportion (approximately 4.95 s). Given that the shortest single trial of our validation experiments was 10 s, our method met the requirements of real-time analysis.

### 3.2. Comparative Analysis of Signal before and after Artifact Removal

**Figure 7:**
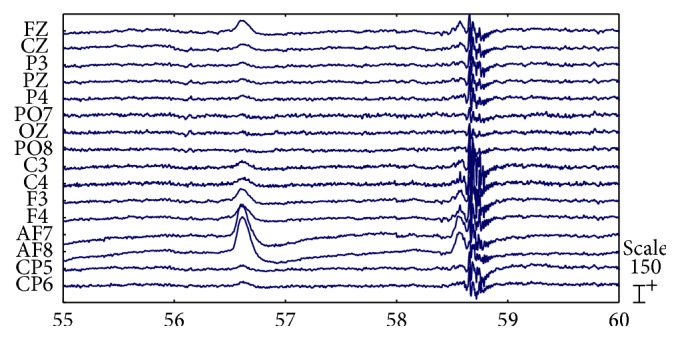
Raw EEG signals with strong EOG and EMG artifacts.

Figure 7 shows a short 16-channel subset of the raw EEG recordings. Strong artifacts caused by eye motion and muscle activity are visible across all channels. The strong artifacts obscure the neural information and are likely to render the corresponding trials useless for the following neural information extraction.

**Figure 8:**
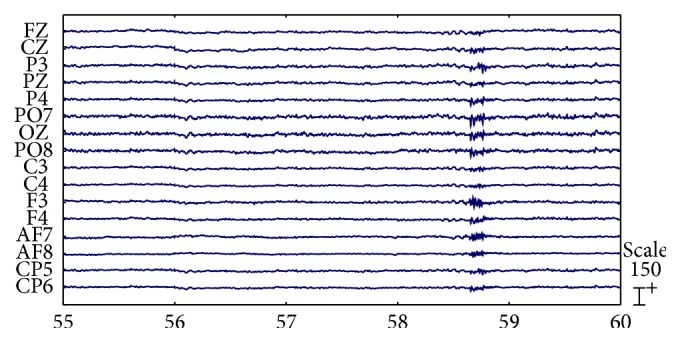
Artifact-removed EEG signals (signals correspond to those depicted in Figure 7).

Figure 8 shows the signals from Figure 7 after artifact removal by our proposed approach. Most of the EOG and EMG artifacts that disturbed the analysis of the raw EEG recordings disappeared. Only small amounts of EMG artifactual activity were still visible. This finding may be due to the removal of only four components with the highest mean correlation scores as the EMG artifacts to prevent excess removal of nonartifact EEG data.

### 3.3. Classification Performances before and after Artifact Removal

#### 3.3.1. Validation 1: Application to Motor Imagery

**Figure 9:**
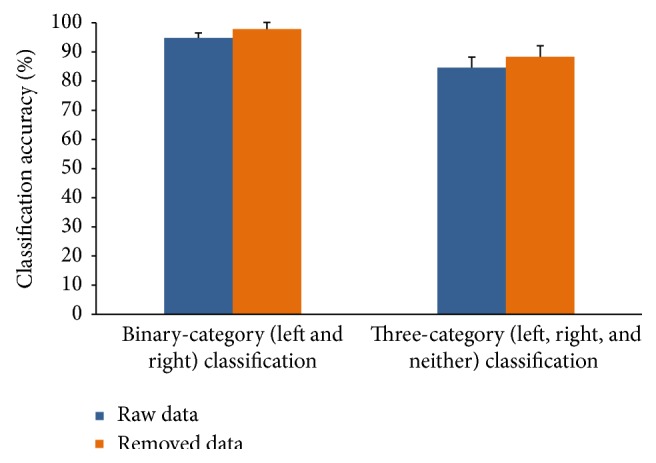
Classification accuracies of raw data and artifact-removed data for binary-category (left and right) and three-category (left, right, and neither) classification. For each subject, an appropriate number of features were selected for the highest accuracy. The mean accuracy was computed across all the subjects. Error bars show the standard deviation of the mean accuracies across all subjects.

All the classification tests in this study were carried out using fivefold cross validation with RBF-kernel SVM. We calculated the offline classification accuracies with different numbers of features selected by different correlation score thresholds. For all subjects, we compared the highest accuracies of the classification between raw data and artifact-removed data. Both the results of binary-category (left and right) classification and three-category (left, right, and neither) classification were utilized to test our method. The mean highest accuracy of binary-category classification across fourteen subjects is shown in Figure 9. For both binary-category and three-category classification, the average prediction accuracy of artifact-removed data was significantly higher than that of raw data on* t*-statistics at a significance level of 0.05. This may be because our proposed method removed the artifact components that influenced the classification, resulting in extracted features that were highly interrelated with the motor imagery task.

#### 3.3.2. Validation 2: Application to Emotion Recognition

**Figure 10:**
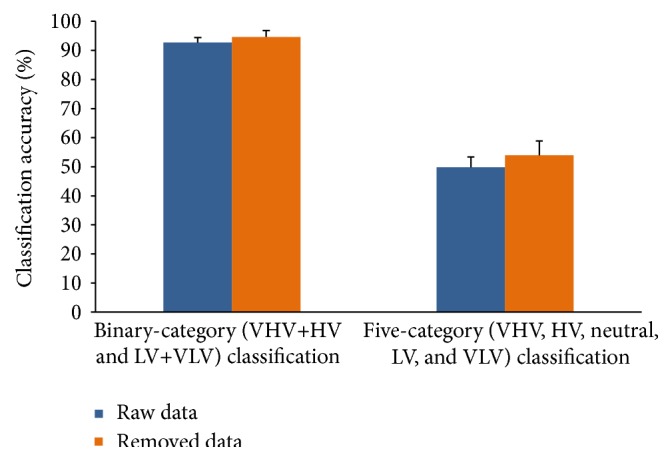
Classification accuracies of raw data and artifact-removed data for binary-category (VHV+HV and LV+VLV) and five-category (VHV, HV, neutral, LV, and VLV) classification. For each subject, an appropriate number of features were selected for the highest accuracy. The mean accuracy was computed among all the subjects. Error bars show the standard deviation of the mean accuracies across all subjects.

In Validation 2, we performed a binary-category (VHV+HV and LV+VLV) classification and five-category (VHV, HV, neutral, LV, and VLV) classification to verify our method. In contrast to Validation 1, features were selected from all the 16 channels, and the maximum number of features was 2880. We also compared the highest accuracies of the classification performances between raw data and artifact-removed data among all subjects (Figure 10). For both binary-category and three-category classification, the average prediction accuracy of artifact-removed data was significantly higher than that of raw data on* t*-statistics at a significance level of 0.05. Thus, the artifact removal method was also effective for EEG data of higher-order cognitive processes. However, we found that the classification performance did not improve or even worsened for some subjects. This finding may be caused by the removed artifact components, which contained some information correlated with emotion, or an involuntary muscle contraction that occurred while the pictures were displayed for emotion induction.

## 4. Discussion and Conclusion

The main idea of our method was to acquire the online a priori artifact information and put them in the WICA with the raw data including artifact to be removed. The artifact components were recognized and removed by sorting the correlation of the marked a priori artifact information and WICs. The a priori artifact information obtained online can effectively reflect the nonneural physiological artifacts during the EEG experiments. And the types of artifacts to be removed were determined by the types of the a priori artifact information. We used eye blinking, eye rolling, and teeth clenching to generate a priori EOG and EMG artifact information, respectively. The EOG artifact produced by eye blinking and eye rolling was mainly contained in two ICs (Figures 5 and 6). This means that the more subtle the acquisition of a priori artifact information is, the more subtle the artifact component may be discriminated. For example, head moving and swallowing may generate the a priori artifact information that can be used to distinguish EMG artifact components mostly related to themselves. Nevertheless, removing too many artifact components may lead to the excess removal of nonartifact EEG data because of the limited total number of WICs (which is not higher than that of channels). In practical applications, which a priori artifact information to acquire should be considered comprehensively based on the number of channels, the influence of the artifact in the experiment, and the burden of the subjects.

There is one thing that needs to be stressed which is that the performance of the chosen ICA method directly determines whether the artifact components can be separated. In this study, we chose WICA by analyzing different methods during preexperiments. Compared with general ICA methods, WICA improves the performance of ICA, since it projects data into a new space where the redundancy is higher and the features of artifacts are fully utilized. The statistical results also demonstrated that it was effective for motor imagery and emotion recognition. However, it cannot be ruled out that other ICA methods may work well in different conditions. Since we only focused on the automatic online artifact removal method in this study, we did not do much research in feature extraction and classification methods, which might affect the classification accuracies more or less.

In this study, a priori artifact information acquired online was introduced into WICA to realize automatic artifact removal for variable subjects and EEG acquisition environments. The proposed method was applied to two experiments, namely, motor imagery and emotion recognition. The statistical results showed that our method significantly improved the classification accuracies for motor imagery and emotion recognition. In addition, our method required no reference channels, massive training samples, and visual inspections, so it was entirely automatic. Therefore, the proposed method may provide an alternative approach for automatic artifact removal, particularly for novice researchers in other fields.

## Figures and Tables

**Table 1:** EEG recording parameters.

EEG recording parameters	

Amplifier	16-channel g.USBamp system (gtec, Graz, Austria)
Sampling frequency	512 Hz
High-pass filter	0.1 Hz
Low-pass filter	60 Hz
Notch filter	50 Hz
Electrode placements	16-channel subset of 10–20 systems (see Figure 7)
Ground	Forehead
Reference	Right earlobe
Electrode material	Ag/AgCl
Recording software	g.Recorder
